# Supplementary material for: Genetic Diversity in Invasive Populations of Argentine Stem Weevil Associated with Adaptation to Biocontrol
Source: Insects. 2020 Jul 14;11(7):441. doi: 10.3390/insects11070441 (PMC7411575; doi:10.3390/insects11070441)
Supplement: Supplementary file 1 [file insects-11-00441-s001.zip › insects-842368-supplementary Figure.pdf]

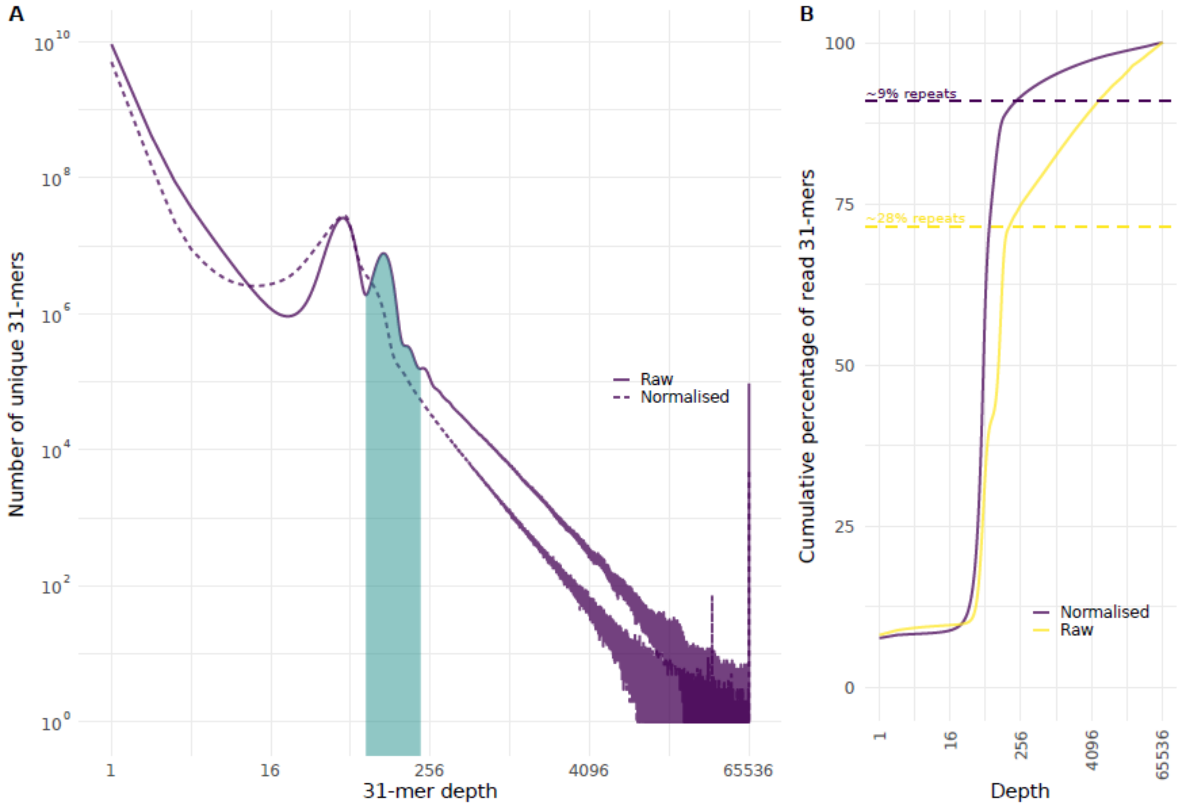

Figure S1. k-mer analysis of the short read sequencing dataset at $k = 31$. **A** k-mer distribution in the short read dataset before and after normalisation. The peak at 84–216× coverage (highlighted in green) suggests a diploid genome with a haploid size of 967 Mb. The haploid peak (22–56× coverage) indicates a high number of polymorphic sites in the single, male individual used to generate this dataset. BBTools [25] estimated 2.1 polymorphisms per 100 bp in this dataset. **B** Cumulative percentage of all k-mers vs. frequency. 72% of all k-mers are at 216× or lower coverage, indicating that the remaining 28% of k-mers are repetitive. The earlier inflexion point at 48% suggests that k-mers in the region from 48–72% may also be from repetitive regions.
