# Supplementary material for: Genetic Diversity in Invasive Populations of Argentine Stem Weevil Associated with Adaptation to Biocontrol
Source: Insects. 2020 Jul 14;11(7):441. doi: 10.3390/insects11070441 (PMC7411575; doi:10.3390/insects11070441)
Supplement: Supplementary file 1 [file insects-11-00441-s001.zip › insects-842368-supplementary Tables.pdf]

Table S1. Repeat content of the subset draft assembly. We used RepeatModeler to detect repeats and the Dfam_3.1 Combined Database with RepeatClassifier to classify them [33,50], then used RepeatMasker to locate repeats in the assembly [35]. 1: Most repeats fragmented by insertions or deletions have been counted as one element.

	Number of elements[1]	Length	Percentage occupied of sequence
Retroelements	208423	102784249 bp	9.24 %
SINEs:	0	0 bp	0.00 %
Penelope	36938	13813895 bp	1.24 %
LINEs:	191930	88657653 bp	7.97 %
CRE/SLACS	0	0 bp	0.00 %
L2/CR1/Rex	63334	24555690 bp	2.21 %
R1/LOA/Jockey	4526	2934064 bp	0.26 %
R2/R4/NeSL	1736	1662589 bp	0.15 %
RTE/Bov-B	13972	4650200 bp	0.42 %
L1/CIN4	0	0 bp	0.00 %
LTR elements:	16493	14126596 bp	1.27 %
BEL/Pao	3775	2519145 bp	0.23 %
Ty1/Copia	344	270624 bp	0.02 %
Gypsy/DIRS1	12155	11090256 bp	1.00 %
Retroviral	0	0 bp	0.00 %
DNA transposons	259238	83605564 bp	7.52 %
hobo-Activator	35507	7694409 bp	0.69 %
Tc1-IS630-Pogo	151555	51159902 bp	4.60 %
En-Spm	0	0 bp	0.00 %
MuDR-IS905	0	0 bp	0.00 %
PiggyBac	4495	1270461 bp	0.11 %
Tourist/Harbinger	9472	1631942 bp	0.15 %
Other (Mirage, P-element, Transib)	2578	1135450 bp	0.10 %
Rolling-circles	64570	11870671 bp	1.07 %
Unclassified:	2562568	583055017 bp	52.41 %
Total interspersed repeats:		769444830 bp	69.17 %
Small RNA:	17929	2803112 bp	0.25 %
Satellites:	0	0 bp	0.00 %
Simple repeats:	135224	7066969 bp	0.64 %
Low complexity:	29913	1422099 bp	0.13 %

Table S2. Number of SNPs under selection using BayeScan [44] (Q < 0.01) or cross-population extended haplotype homozygosity (XPEHH) analysis [51,52] ($-\log_{10}p > 4$). α is BayeScan's locus-specific component of F_{ST} coefficient [44]. Positive values suggest diversifying selection. Positive XPEHH scores suggest selection in the South group, and negative scores suggest selection in the North group.

Contig	Total SNPs	BayeScan SNPs	BayeScan region	α	XPEHH SNPs	XPEHH region	XPEHH

contig_40523	26	5	103,989 – 111,755	1.93 – 2.14	0		
contig_11164	23	4	60,487 – 179,797	1.84 – 2.05	0		
scaffold_43207	11	4	102,783 – 102,811	1.70 – 2.14	0		
contig_12006	10	3	46,975 – 47,031	2.09 – 2.15	0		
contig_13287	10	3	58,727 – 58,738	1.68 – 1.98	0		
contig_18336	20	3	233,287 – 342,618	1.90 – 1.99	0		
contig_2677	22	3	109,260 – 110,069	1.79 – 1.99	0		
contig_39072	10	3	55,060 – 55,126	2.06 – 2.14	0		
contig_23638	6	2	118,602 – 118,616	2.08 – 2.15	0		
contig_37676	17	2	47,912 – 47,954	2.01 – 2.03	0		
contig_4080	13	2	26,024 – 26,027	1.78 – 1.79	0		
contig_1196	24	1	393,177	1.68	0		
contig_12091	3	1	43,431	2.02	0		
contig_14933	7	1	80,478	1.98	0		
contig_19450	6	1	37,951	2.39	0		
contig_202	8	1	80,251	2.03	0		
contig_20252	20	1	34,278	1.87	0		
contig_205	6	1	28,713	2.13	0		
contig_21253	6	1	43,048	1.76	0		
contig_23312	12	1	171,714	1.95	0		
contig_27115	20	1	111,838	1.94	0		
contig_28985	6	1	22,034	2.01	0		
contig_3057	13	1	272,534	1.91	0		
contig_8456	36	1	88,819	1.92	0		
contig_2544	65	0			3	83,144 – 83,595	-6.46 – -5.23
